# Supplementary material for: Indirect COVID-19 health effects and potential mitigating interventions: Cost-effectiveness framework
Source: PLoS One. 2022 Jul 18;17(7):e0271523. doi: 10.1371/journal.pone.0271523 (PMC9292069; doi:10.1371/journal.pone.0271523)
Supplement: S1 File — (DOCX) [file pone.0271523.s001.docx]

**Supplement A: BRACE model detailed analyses**

We applied the BRACE modeling approach to six different priority public health conditions for which there is evidence of increased prevalence during the pandemic: adult depressive symptoms, excessive alcohol use, opioid use disorder (OUD), homelessness, intimate partner violence (IPV), and stroke mortality. “Tornado” diagrams were created to summarize the one-way sensitivity analyses (SAs). These figures indicate how two key outcomes – net overall cost and QALYs gained – varied according to uncertainty in the values of the most influential health and cost inputs. As with the multivariate analyses, these analyses suggested that the results are qualitatively robust – usually net savings (i.e., “dominant”) and substantial QALY gains. A full set of one-way and multivariate SAs is available as a technical supplement on request.

Below, we provide a detailed discussion of the results for adult depressive symptoms, as a case study, to illustrate the range of findings possible using this approach.

**Note:** We did not attempt to model racial/ethnic disparities due to concerns about the poor quality of current data. For example, for IPV, studies reported lower growth in police reports by Black women compared with White and Hispanic women. Data on IPV reporting from the pre-COVID-19 period data suggested that IPV may be 60% higher among Hispanic women and 90% higher among Black women compared to White women. If this difference in pre-COVID-19 rates led to larger differences in the rise in IPV rates due to COVID-19, the economic analyses will find that per capita health and economic burdens will be higher (e.g., 60% greater among Hispanics than Whites), as will intervention costs and benefits. The cost-effectiveness ratio would stay the same. In fact, post-COVID-19 increases in IPV may be even greater among Black and Hispanic women due to the heightened exposure of ethnic minority families to the economic fallout of the pandemic – an extreme stressor on families, which could increase IPV risk.

**Depressive symptoms (case study)**

Our BRACE model analysis of adult depressive symptoms (including depressive symptoms not necessarily meeting formal diagnostic criteria for depression) is presented in Table A1. Input values were derived from the most relevant studies in our literature review (Table A2). Due to imperfect internal validity (study precision and accuracy for study subjects) and external validity (applicability to COVID-19 in California), we conducted extensive sensitivity analyses to quantify the impact of uncertainty in input values on results.

Prevalence of depressive symptoms prior to COVID-19 was estimated at 18.5%. Direct medical costs (all costs inflated to 2020) were estimated at $12,806 per case in the short term, and non-medical direct costs at $1,130. With a recurrence risk of 45% and estimated four episodes of depression, if there is recurrence, the anticipated long-term costs were approximately twice as high as short-term. Discounted at 3%, total direct cost per episode was $32,599.

Morbidity was quantified in health state utility (0 to 1, where 1 indicates perfect health) and was incorporated into quality-adjusted life years (QALYs). Studies suggested a 0.5 drop in utility during the episode, which persists for 0.5 years, the mean duration of a depressive episode. The drop was similar in subsequent episodes if they occurred (45%), and we spread these drops in utility over two years. Mortality, largely due to suicide, was estimated at 4 per 1,000 in the short-term and similar risk for multiple subsequent episodes.

COVID-19 increased the risk of depressive symptoms by an estimated 37% (median of identified studies). This increased risk resulted in a depressive symptom prevalence of 25.3%, of which 6.9 percentage points were due to COVID-19. The resulting added direct cost per capita was $2,231, and there were 0.069 lost QALY per capita.

We examined one intervention that could mitigate the effects of increases in adult depressive symptoms: cognitive-behavioral therapy with selective serotonin reuptake inhibitor medications (CBT + SSRI). This intervention, like all examined in the BRACE model, is not COVID-19-specific, for three reasons. First, the intervention was designed and tested before COVID-19; we found no reports of COVID-19-adapted versions. Second, we believe it is challenging to definitively attribute a specific case of depression or other conditions we examined to COVID-19. A detailed clinical assessment *might* suggest a likelihood of COVID-19 etiology, e.g., if depressive symptoms start soon after a job layoff due to COVID-19, but the determination would not be definitive in the presence of other risk factors and related medical history. Third, we could identify no compelling reason to limit interventions to those individuals who appear more likely to have COVID-19 as a substantial contributing cause; rather, we believe that interventions will be tailored to diagnosis, whatever the etiology.

CBT + SSRI had an estimated cost of $878 per client, with 25% efficacy. Assuming 20% coverage in a population of one million, the cost was $45 million. This led to a 1.3 percentage point reduction in prevalence (from 25.3% to 24.1%), with 12,707 QALYs gained. The savings (averted direct costs) were estimated at $413 million, yielding net savings of $61 million within a year and $241 million by 10 years. By traditional economic criteria, the intervention appeared quite promising, i.e., both mitigating indirect health harms of COVID-19 and producing substantial economic savings, even in the first year.

| **Table A1. BRACE results for effects of COVID-19 on depressive symptoms in adults** |
| --- |


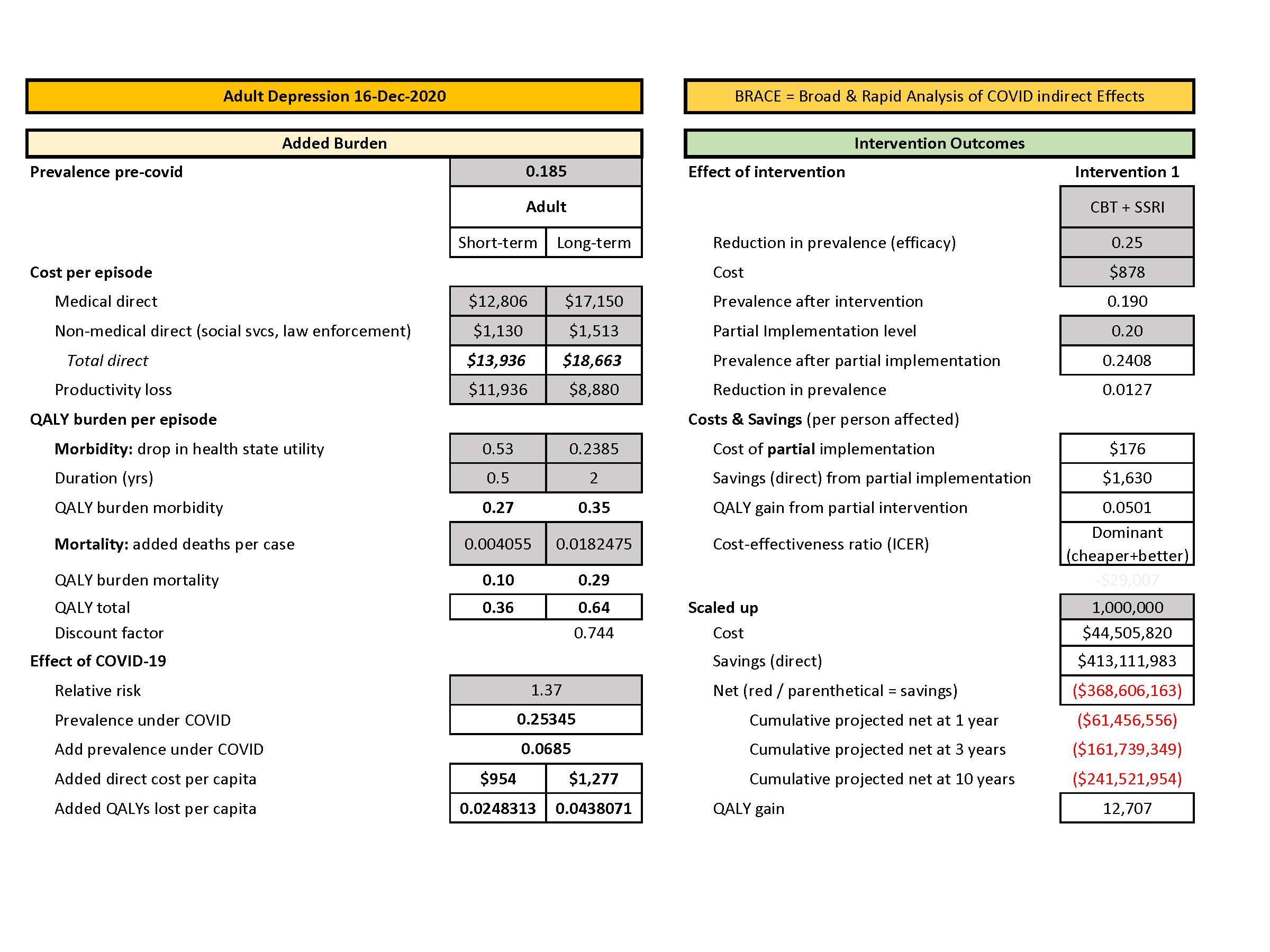


***Sensitivity analyses***

We conducted one-way and multivariate sensitivity analyses (SA) for all results to address uncertainty in key inputs. Projections were conducted assuming an eligible population of one million individuals and 20% intervention coverage of the affected population. In multivariate analyses, we varied all input variables based on defined probability distributions and ranges. In one-way sensitivity analyses, each input was varied individually while all others were kept constant. The values included in the sensitivity analyses were obtained through a robust review of the literature. In cases where a reliable value could not be identified, expert opinion was used to inform input values.

Based on one-way SA for depressive symptoms, costs related to implementing CBT + SSRI were most impacted by variation in cost of the intervention itself, followed by the prevalence of depressive symptoms under COVID-19. Net savings associated with CBT + SSRI were most influenced by the effectiveness of this intervention, prevalence of depressive symptoms before COVID-19, and medical cost per episode of depressive symptoms. Shown below in Figures A1 and A2 are the one-way SA for net savings and QALY gain per million with CBT + SSRI.

A separate one-way analysis on the proportion of short-term costs that are incurred in year 1 (60% at base case) showed that if this value was 30%, year 1 savings were reduced to $8.5 million. If this value was instead 90%, over $114 million could be saved within one year.

**Figure A1. One-way sensitivity analyses for net savings per million population with cognitive-behavioral therapy with selective serotonin reuptake inhibitor medications (CBT + SSRI) intervention for adult depressive symptoms**

**
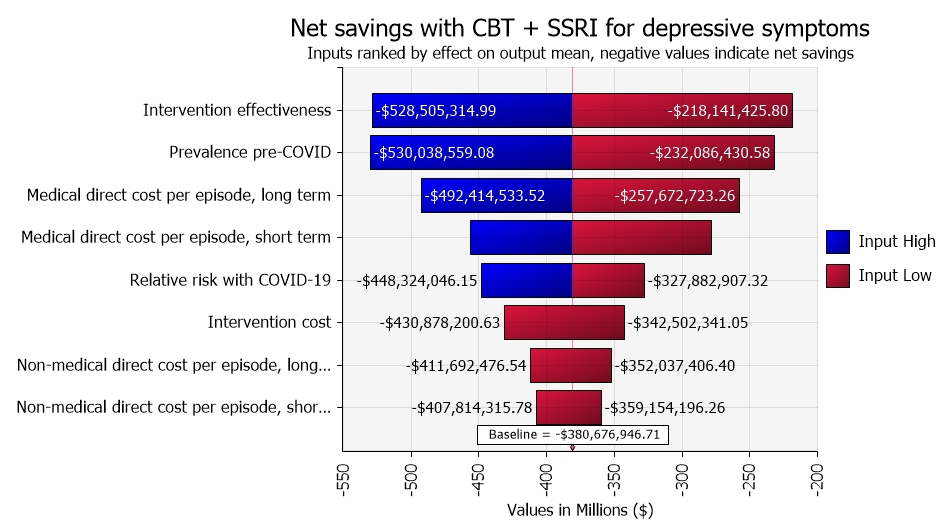
**

Net savings ranged from $218 to $528 million per million population in a one-way SA for CBT + SSRI.

**Figure A2. One-way sensitivity analyses for quality-adjusted life years (QALYs) gained per million population with cognitive-behavioral therapy with selective serotonin reuptake inhibitor medications (CBT + SSRI) intervention for adult depressive symptoms**


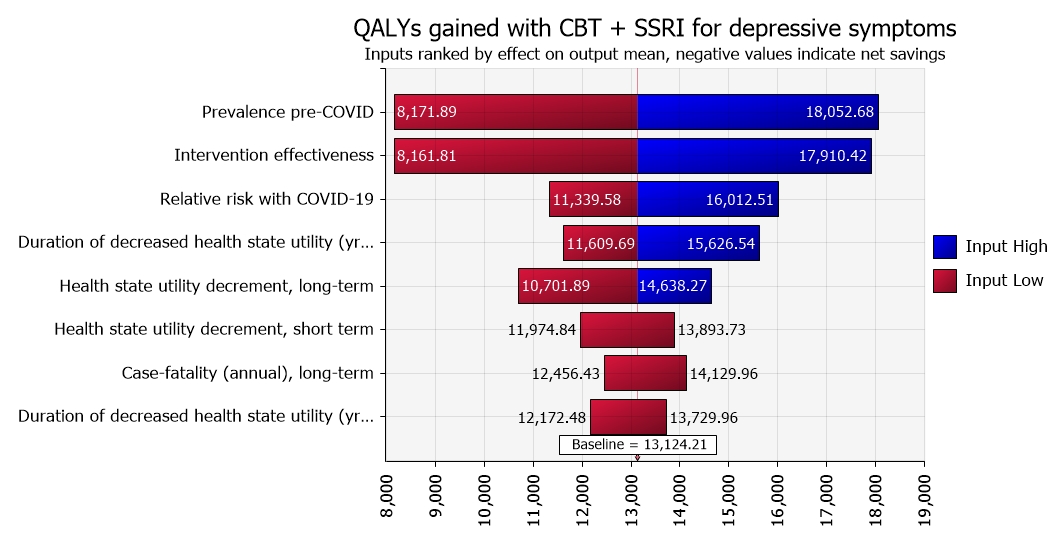


QALYs gains ranged from 8,000 to 18,000 per million population in a one-way SA for CBT + SSRI.

***Overview of findings***

Our analysis of adult depressive symptoms is presented in full detail above. We estimated a 37% increase in this condition due to COVID-19, resulting in a prevalence near 25%. The cost per case, including short-term and long-term consequences in the affected adult was nearly $32,000. The cost per case translates to a COVID-19-associated added direct cost per capita of over $2,200. CBT + SSRI medications had an estimated cost of $878 per case, with 25% effectiveness. Assuming 20% coverage in a population of one million, the cost was $45 million. This intervention led to a 1.3 percentage point reduction in prevalence (from 25.3% to 24.1%), with 12,700 QALYs gained. The savings (averted direct costs) were estimated at $413 million, yielding net savings of $61 million within a year and $241 million by 10 years. By traditional economic criteria, this finding makes the intervention quite promising, i.e., both mitigating indirect health harms of the pandemic and producing substantial economic savings for California.

**Table A2. Inputs, uncertainty ranges, and sources for depression BRACE model**

| **Input** | **Base-case value** | **Uncertainty range** | **Sources and remarks** |
| --- | --- | --- | --- |
| Prevalence before COVID-19 | 18.5% | 9.25% - 27.8% | Villaroel and Terlizzi 2020 |
| Risk ratio for prevalence after COVID-19 | 1.37 | 1.20 – 2.56 | Pierce 2020, Smalley 2020, Twenge and Joiner 2020 |
| ***Costs**** | | | |
| Short-term medical | $12,806 | $3,726 - $21,925 | Greenberg 2015, Olfson 2018. Includes inpatient, emergency department, outpatient medical services; prescription drugs; other medical costs. |
| Long-term medical | $23,051 | $11,525 - $34,576 | Greenberg 2015, Olfson 2018. Assumed 45% risk of recurrence and maximum 4 episodes, with same costs as short-term medical. |
| Short-term non-medical | $1,130 | $565 - $1,695 | Greenberg 2015. Disability cost to workplace. |
| Long-term non-medical | $2,034 | $1,017 - $3,051 | Greenberg 2015. Assumed 45% risk of recurrence and maximum 4 episodes, with same costs as long-term non-medical. |
| ***Health*** | | | |
| Short-term health state utility decrement | 0.53 | 0.50 – 0.56 | Sobocki 2007 |
| Long-term health state utility decrement | 0.24 | 0.12 – 0.36 | Sobocki 2007. Assumed 45% risk of recurrence. |
| Short-term mortality | 0.4% | 0.2% – 0.6% | Conner 2019, Hasin 2018 |
| Long-term mortality | 1.8% | 0.9% – 2.7% | Conner 2019, Hasin 2018. Assumed 45% risk of recurrence. |
| ***Intervention: Cognitive behavioral therapy and SSRI medication*** | | | |
| Cost | $878 | $92 - $1,664 | California Deparment of Health Care Services 2021 |
| Effectiveness | 25% | 12.5% - 37.5% | Arroll 2009 |

*Costs are in 2020 US dollars. Long-term costs are presented before discounting.

**Intimate partner violence**

We estimated an 11% increase in this condition due to COVID-19 (Table A3), resulting in a prevalence of 6.1%. The cost per case, including short-term and long-term consequences in the affected adult, was $116,000; this cost per case translates to a COVID-19-associated added direct cost per capita of $700. Nurse-family partnership (NFP) home visiting program had an estimated cost of $13,000 per family, with 21% effectiveness. Assuming 20% coverage in a population of one million, the cost was $165 million. This intervention, if implemented, was projected to reduce the prevalence of IPV by approximately 0.3 percentage points (to 5.8%), with 22,000 QALYs gained. The savings (averted direct costs) were estimated at $296 million, yielding net costs of $70 million within a year and net savings of $55 million by 10 years. The results highlight that implementing this intervention may not only mitigate indirect health harms of COVID-19 but also produce substantial economic savings. If only 30% of short-term costs were incurred in year 1 (as opposed to 60% in base case), year 1 net costs were greater at $117 million. If this value was instead 90%, the intervention would have a net cost of $22 million in the first year.

**Table A3. BRACE results for effects of COVID-19 on intimate partner violence**

**
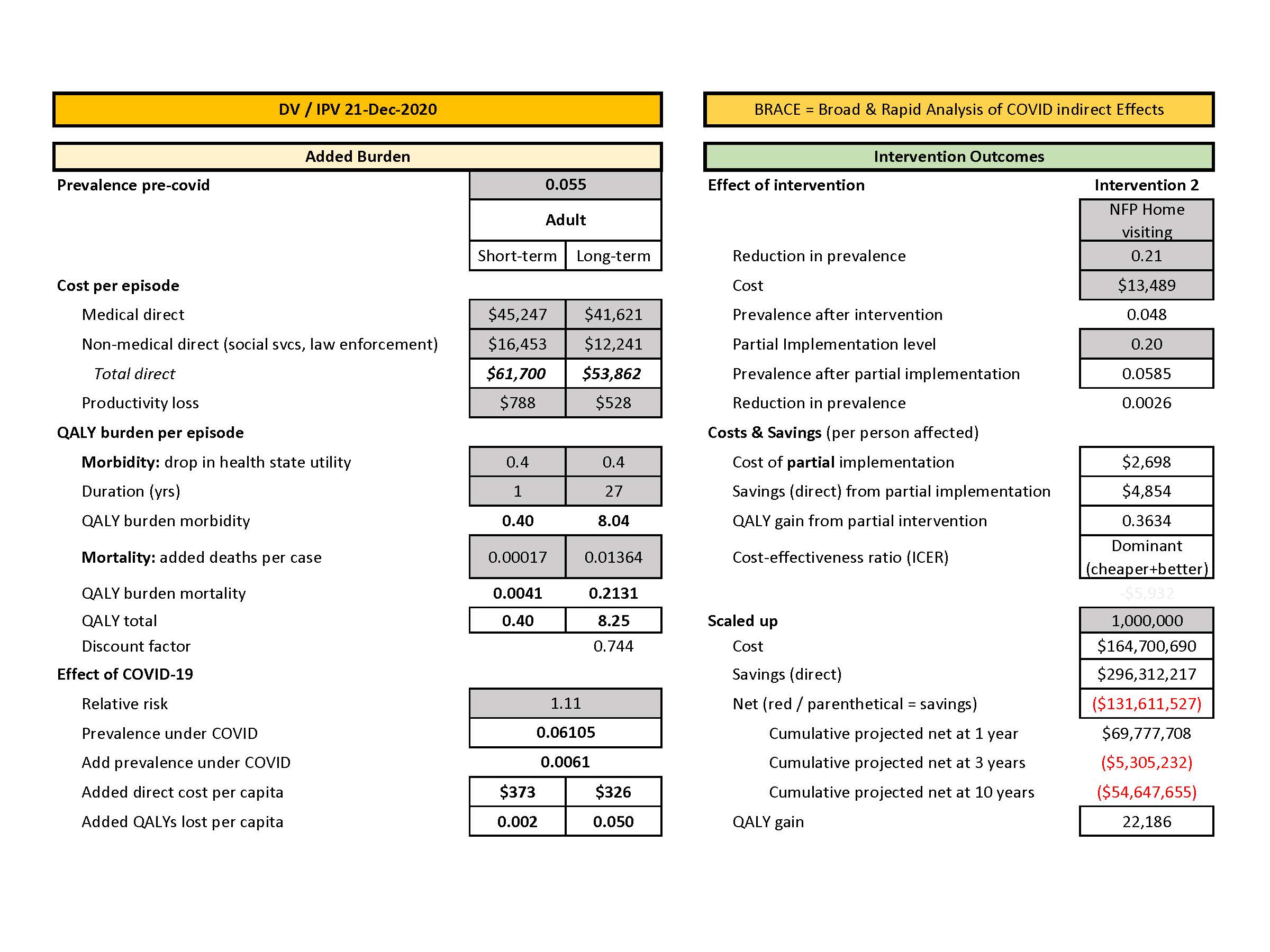
**

**Figure A3. One-way sensitivity analyses for net savings with nurse-family partnership for intimate partner violence (IPV)**


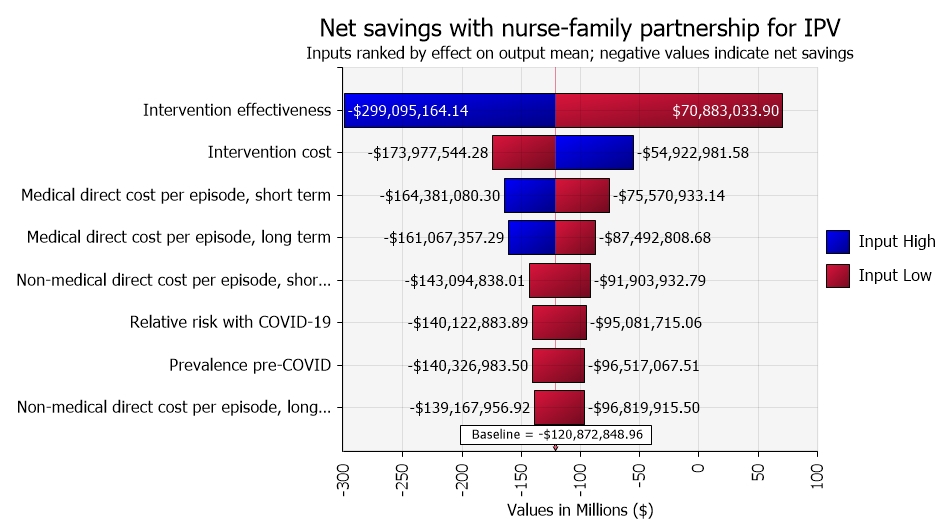


The nurse-family partnership intervention yielded net savings up to $300 million or net costs up to $71 million per million population in a one-way SA.

**Figure A4. One-way sensitivity analyses for quality-adjusted life years (QALYs) gained with nurse-family partnership for intimate partner violence (IPV)**


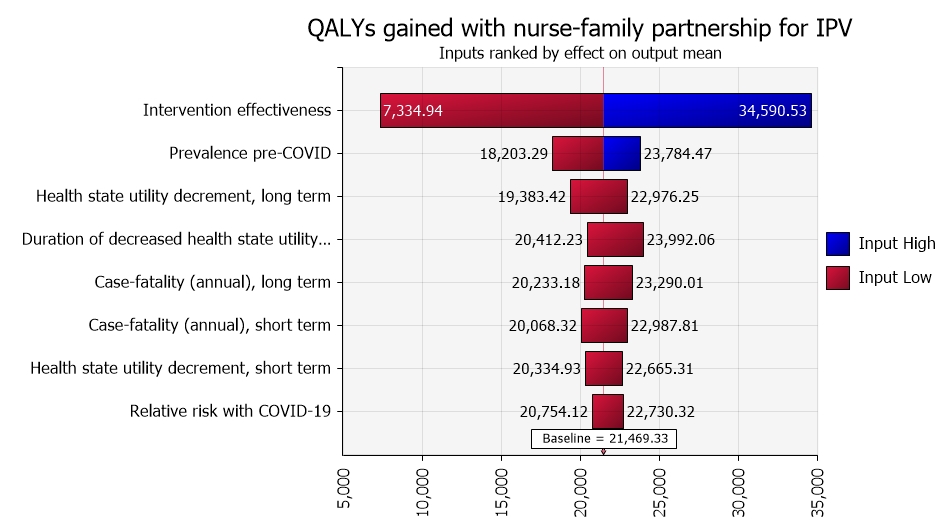


QALY gains ranged from 7,300 to 35,000 per million population in a one-way SA with nurse-family partnership intervention.

**Table A4. Inputs, uncertainty ranges, and sources for intimate partner violence BRACE model**

| **Input** | **Base-case value** | **Uncertainty range** | **Sources and remarks** |
| --- | --- | --- | --- |
| Prevalence before COVID-19 | 5.5% | 4.8% - 6.3% | Smith 2018 |
| Risk ratio for prevalence after COVID-19 | 1.11 | 1.05 – 1.16 | Bullinger 2020, Mohler 2020, Piquero 2020 |
| ***Costs**** | | | |
| Short-term medical | $45,247 | $22,623 - $67,870 | Peterson 2018. Includes social service in the emergency room, emergency room cost, hospitalization, rape kit, physical assault and stalking victimization. |
| Long-term medical | $55,942 | $27,971 - $83,913 | Peterson 2018. Lifetime cost including long-term mental health, substance use, and physical health conditions. |
| Short-term non-medical | $16,453 | $8,227 - $24,680 | Peterson 2018. Includes police protection, judicial, and legal costs. |
| Long-term non-medical | $16,453 | $8,227 - $24,680 | Peterson 2018. Assumed equal to short-term non-medical cost. |
| ***Health*** | | | |
| Short-term health state utility decrement | 0.4 | 0.2 – 0.6 | Wittenberg 2006 |
| Long-term health state utility decrement | 0.4 | 0.36 – 0.44 | Ferrari 2014 |
| Short-term mortality | 0.017% | 0.01% - 0.02% | Peterson 2018 |
| Long-term mortality | 1.36% | 0.74% - 2.08% | Chandan 2020 |
| ***Intervention: Nurse-family partnership home visiting*** | | | |
| Cost | $13,489 | $6,745 - $20,234 | Wu 2017 |
| Effectiveness | 21% | 1% - 36% | Miller 2015 |

*Costs are in 2020 US dollars. Long-term costs are presented before discounting.

**Homelessness**

Nearly seven-fold increase is projected in this condition due to COVID-19, resulting in a prevalence of 2.5% (Table A5). The cost per case, including short-term and long-term consequences in the affected adult, was $100,000, resulting in a COVID-19-associated added direct cost per capita of $2,150. Rent subsidies had an estimated cost of $9,000 per client, with 43% effectiveness in reducing consequences (accounting for intervention delivery after experiencing homelessness). Assuming 20% coverage in an eligible population of one million, the cost was $47 million. This led to a reduction in prevalence of homelessness to 2.3%, with 1,600 QALYs gained. The savings (averted direct costs) were estimated at $215 million, yielding net costs of $5 million within a year and net savings of $92 million by 10 years. Thus, the intervention appears to be dominant, or achieves a cost-savings. If the increase in homelessness were half as much as observed after the 2008 Recession, the intervention led to fewer net savings ($67 million overall, $35 million by 10 years), and remained dominant as 1291 QALYs were gained. If only 30% of short-term costs were incurred in year 1 (as opposed to 60% in base case), the intervention would have a net cost of $26 million in year one. If this value was instead 90%, the intervention would yield net savings of over $16 million within one year.

**Table A5. BRACE results for effects of COVID-19 on housing insecurity**


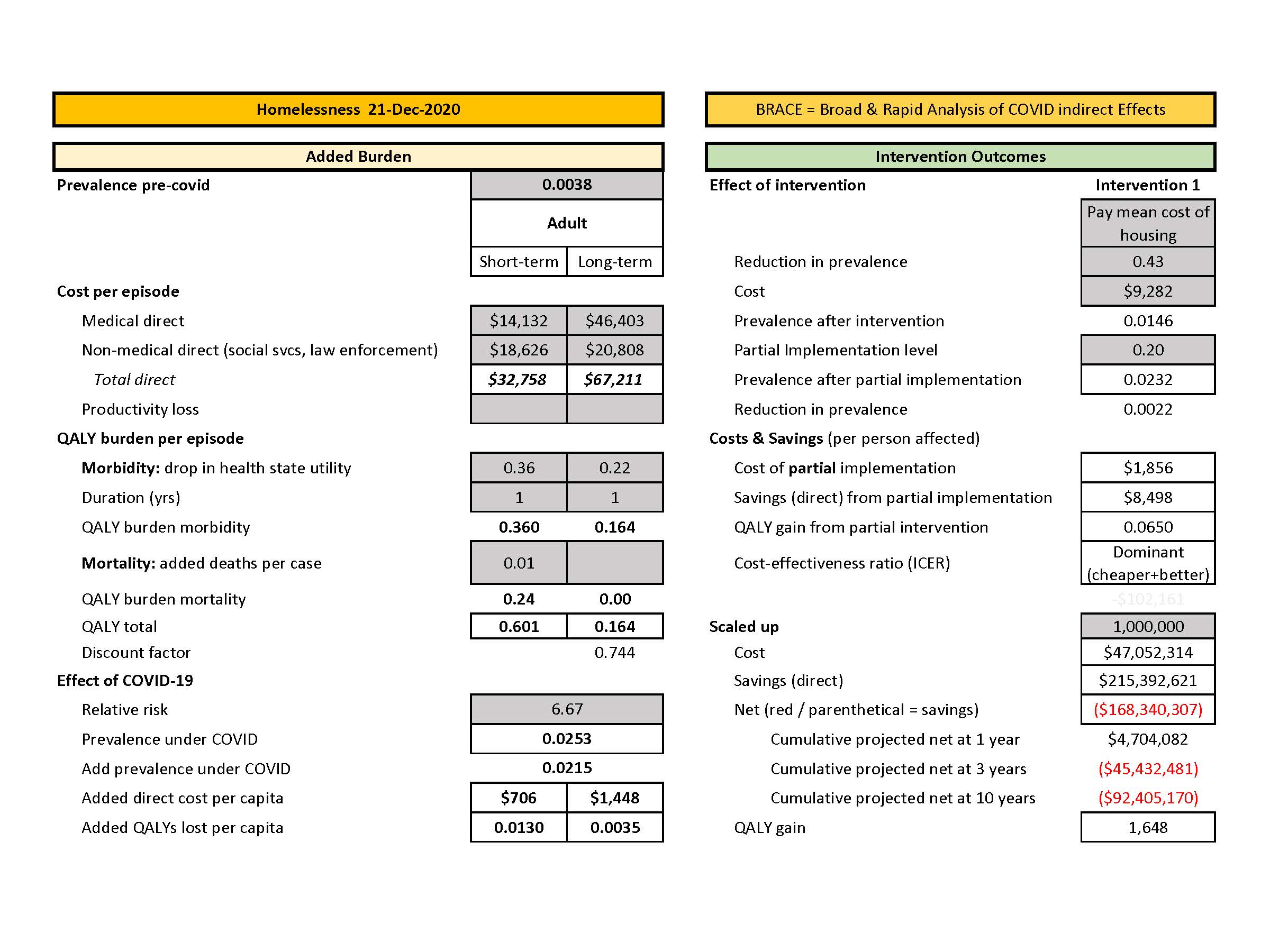


**Figure A5. One-way sensitivity analyses for net savings with rent subsidies for homelessness**


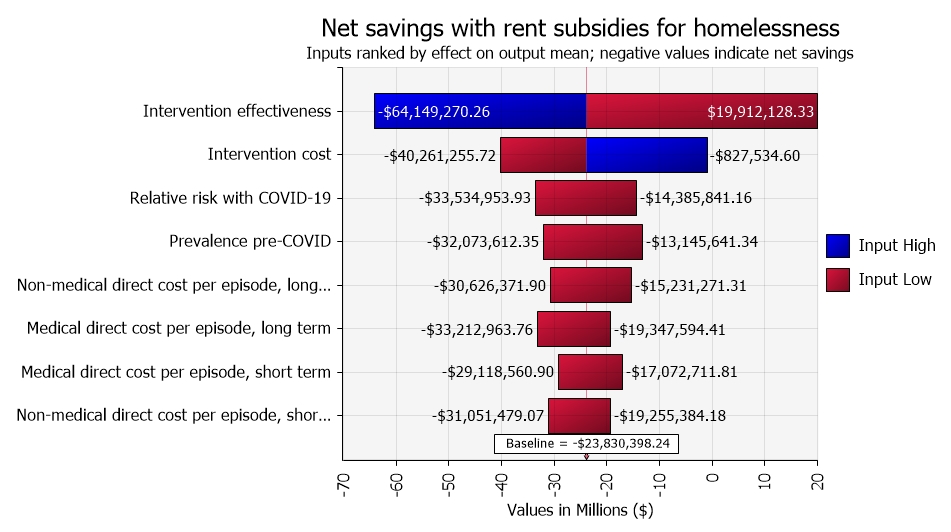


The rent subsidy intervention yielded net savings up to $64 million or net costs up to $20 million per million population in a one-way SA.

**Figure A6. One-way sensitivity analyses for quality-Adjusted Life Years (QALYs) gained with rent subsidies for homelessness**

**
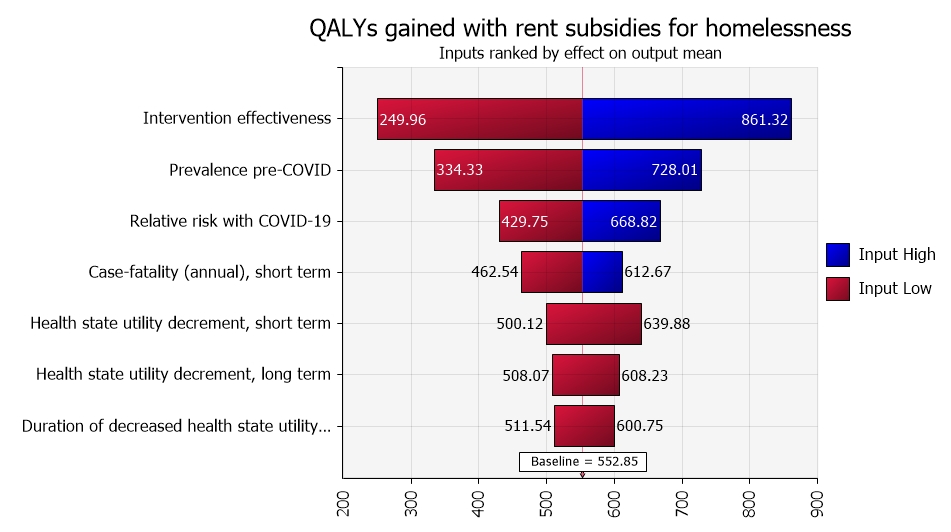
**

QALY gain ranged from 250 to 860 per million population in a one-way SA for rent subsidies.

**Table A6. Inputs, uncertainty ranges, and sources for homelessness BRACE model**

| **Input** | **Base-case value** | **Uncertainty range** | **Sources and remarks** |
| --- | --- | --- | --- |
| Prevalence before COVID-19 | 0.38% | 0.19% - 0.57% | United States Census Bureau 2020, United States Interagency Council on Homelessness 2020 |
| Risk ratio for prevalence after COVID-19 | 6.67 | 5.34 – 8.00 | Institute for Children 2013 |
| ***Costs**** | | | |
| Short-term medical | $14,132 | $7,066 - $21,198 | Wu and Stevens 2016. Los Angeles County Department of Health Services and Department of Mental Health estimate per instance of homelessness. |
| Long-term medical | $62,369 | $45,809 - $78,929 | Zaretzky 2013. Assumed 10% will remain homeless; lifetime healthcare cost. |
| Short-term non-medical | $18,626 | $9,313 - $27,939 | Wu and Stevens 2016. Los Angeles County Departments of Public Health, Social Services, Sheriff, and Probation estimate. |
| Long-term non-medical | $27,968 | $18,960 - $36,976 | Zaretzky 2013. Assumed 10% will remain homeless. Lifetime judicial cost and cost of placing child in care. |
| ***Health*** | | | |
| Short-term health state utility decrement | 0.36 | 0.32 – 0.4 | Kozloff 2019 |
| Long-term health state utility decrement | 0.22 | 0.16 – 0.34 | Kozloff 2019. Duration was limited to 1 year. |
| Short-term mortality | 1% | 0.5% - 1.5% | Los Angeles County Department of Public Health 2019. Assumed ¼ of traditional homelessness. |
| Long-term mortality | 0% | n/a | Assumed no long-term mortality due to COVID-19-related homelessness. |
| ***Intervention: Rent subsidy*** | | | |
| Cost | $9,282 | $4,641 - $13,923 | Basu 2012, Gubits 2016, Larimer 2009, McLaughlin 2011, United States Census Bureau 2020, Wright 2016 |
| Effectiveness | 43% | 21.5% - 64.5% | Assumed take-up offsets 50% of increased risk due to COVID-19. |

*Costs are in 2020 US dollars. Long-term costs are presented before discounting.

**Opioid use disorder**

A 63% increase was expected in this condition due to COVID-19, resulting in a prevalence of 1.8% (Table A7). The cost per case, including short-term and long-term consequences in the affected adult, was $80,000, resulting in a COVID-19-associated added direct cost per capita of $551. Medication-assisted treatment (MAT) with methadone or buprenorphine had an estimated cost of nearly $16,000 per client, with 39% effectiveness in reducing negative health and financial (e.g., medical and non-medical direct cost) consequences. Assuming 20% coverage in an eligible population of one million, the cost was $56 million. This intervention, if implemented, was estimated to lead to 0.14 percentage points reduction in the prevalence of opioid use disorder (to about 1.7%), with 5,600 QALYs gained. The savings (averted direct costs) were estimated to be $111 million, with $36 million net costs in the first year and net savings of $15 million by 10 years. If only 30% of short-term costs were incurred in year 1 (as opposed to 60% in base case), the intervention would have a net cost of $46 million in year one. If this value was instead 90%, the year one net costs would be reduced to $26 million.

**Table A7. BRACE results for effects of COVID-19 on opioid use disorder**

**
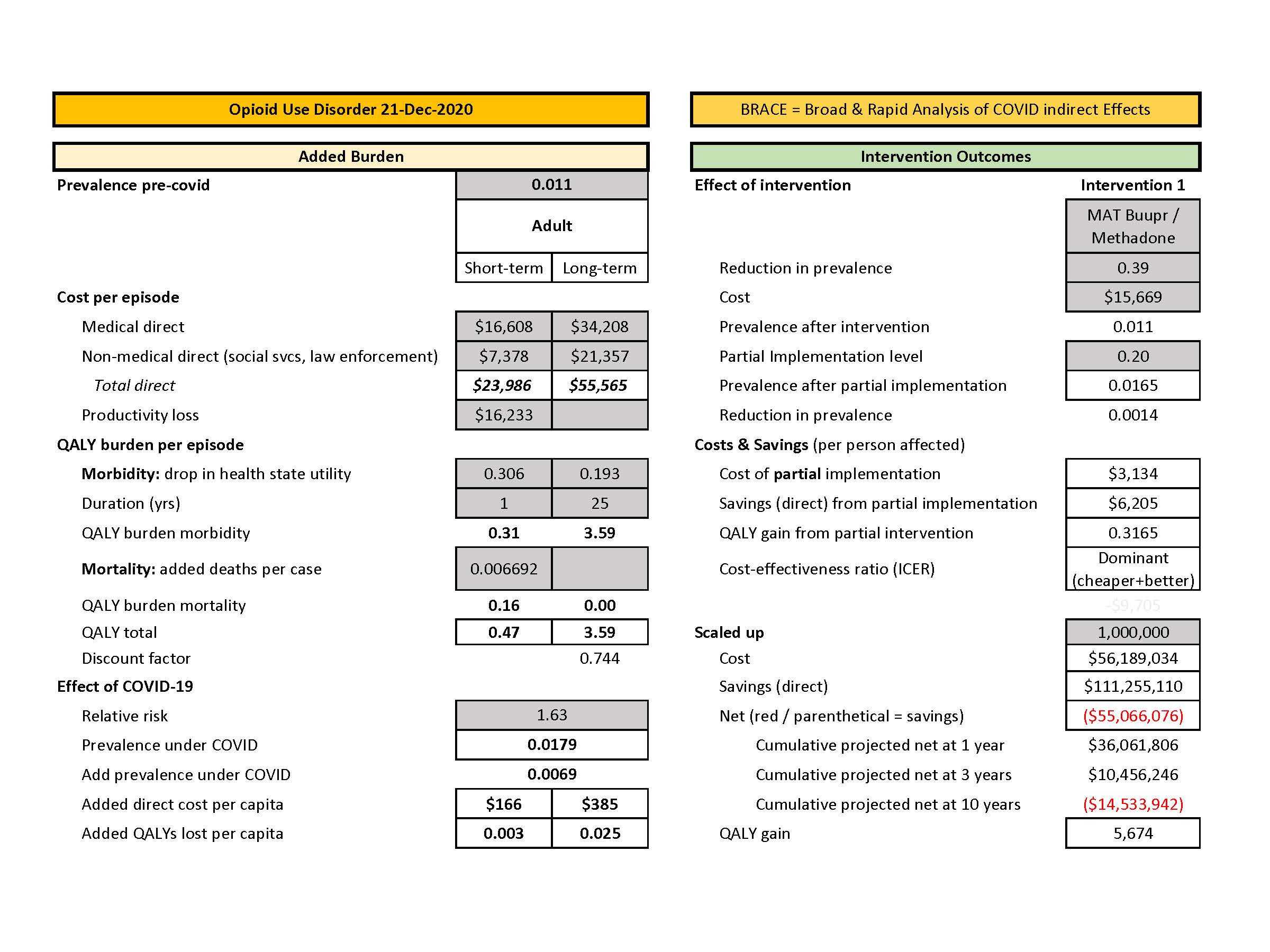
**

**Figure A7. One-way sensitivity analyses for net savings medication-assisted treatment for opioid use disorder**


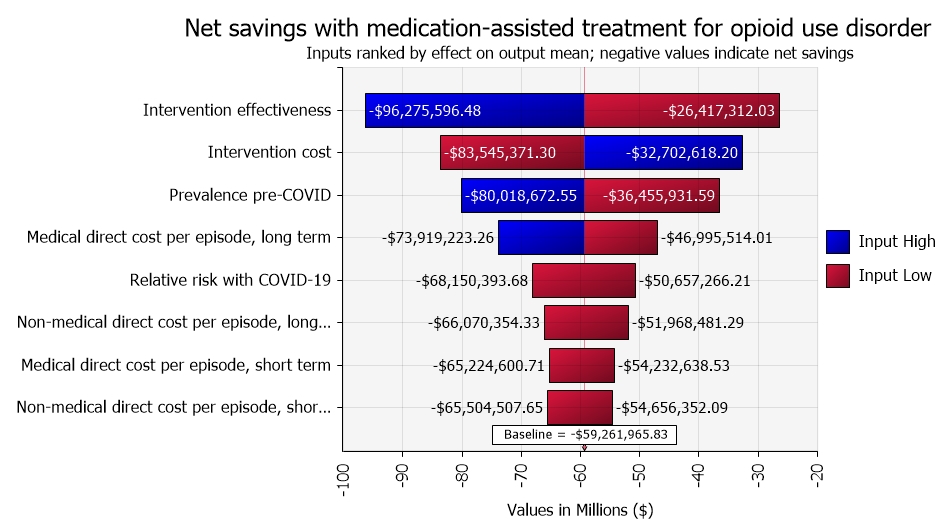


The intervention appeared to yield net savings of $26 to 96 million per million population in one-way SAs.

**Figure A8. One-way sensitivity analyses for quality-adjusted life years (QALYs) gained with medication-assisted treatment for opioid use disorder**


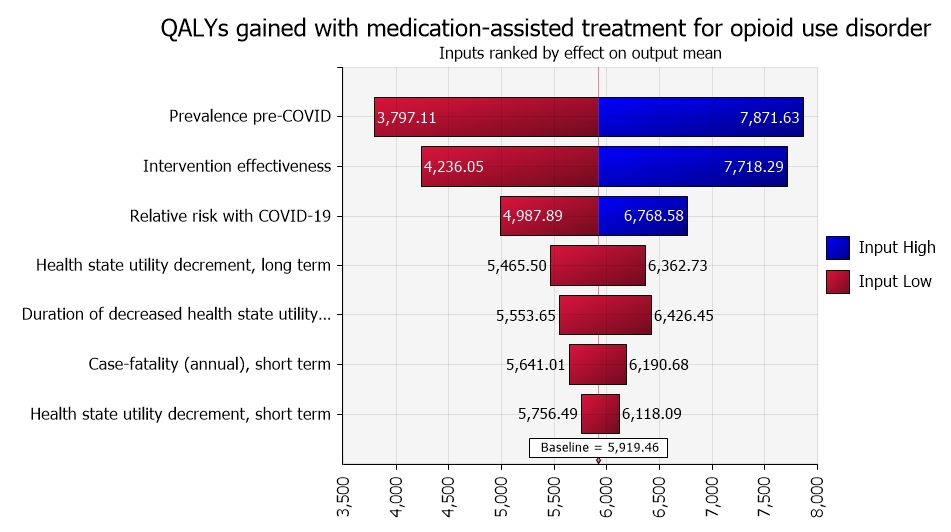


QALY gain ranged from 3,700 to 7,800 per million population in one-way SAs.

**Table A8. Inputs, uncertainty ranges, and sources for opioid use disorder BRACE model**

| **Input** | **Base-case value** | **Uncertainty range** | **Sources and remarks** |
| --- | --- | --- | --- |
| Prevalence before COVID-19 | 1.10% | 0.55% - 1.65% | Center for Behavioral Health Statistics and Quality 2020 |
| Risk ratio for prevalence after COVID-19 | 1.63 | 1.33 – 1.98 | Ahmed 2020, Glober 2020 |
| ***Costs**** | | | |
| Short-term medical | $16,608 | $14,019 - $ 20,170 | Florence 2021, Kirson 2017. Includes costs from private insurance, Medicare, Medicaid, Veteran’s Association, Other, and uninsured costs for non-fatal cases. |
| Long-term medical | $45,978 | $30,666 - $61,290 | Krebs 2018. Healthcare utilization cost for relapse (data unavailable for long-term effect of opioid use). |
| Short-term non-medical | $7,378 | $7,061 - $7,698 | Florence 2021. Includes criminal justice (police protection, legal and adjudication, correctional facilities, property loss due to crime) for non-fatal cases. |
| Long-term non-medical | $28,706 | $21,991 - $35,421 | Bansback 2018. Estimated lifetime cost of involvement in violent and property crimes, criminal charges for any crime (including costs borne by the criminal justice system and out-of-pocket costs from victimization). |
| ***Health*** | | | |
| Short-term health state utility decrement | 0.31 | 0.27 – 0.34 | Wittenberg 2016 |
| Long-term health state utility decrement | 0.19 | 0.17 – 0.22 | Wittenberg 2016 |
| Short-term mortality | 0.70% | 0.35% - 1.04% | Krawczyk 2020 |
| Long-term mortality |  |  |  |
| ***Intervention: Screening and brief intervention*** | | | |
| Cost | $15,669 | $6,254 - $25,084 | Larochelle 2020, The Council of Economic Advisors 2017 |
| Effectiveness | 39% | 26% - 58% | Ma 2019 |

*Costs are in 2020 US dollars. Long-term costs are presented before discounting.

**Excessive Alcohol Use**

A 19% increase in this condition was estimated due to COVID-19, resulting in a prevalence of 7.3% (Table A9). The estimated cost per case, including short-term and long-term consequences in the affected adult was $94,000, resulting in a COVID-19-associated added direct cost per capita of $1,090. A screening and brief alcohol intervention (SBIRT) had an estimated cost of $450 per client, with 15% effectiveness in reducing consequences. Assuming 20% coverage in an eligible population of one million, the cost was $6.5 million. This intervention, if implemented, was estimated to reduce the prevalence by 0.2 percentage points (to about 7.0%), with 4,800 QALYs gained. The savings (averted direct costs) were estimated at $205 million, making the intervention “dominant,” with net savings of $9 million within a year and $107 million by 10 years. If only 30% of short-term costs were incurred in year 1 (as opposed to 60% in base case), year one net savings would be reduced to $1.2 million. If this value was instead 90%, the year one net savings would be nearly $17 million.

**Table A9. BRACE results for effects of COVID-19 on excessive alcohol use**

**
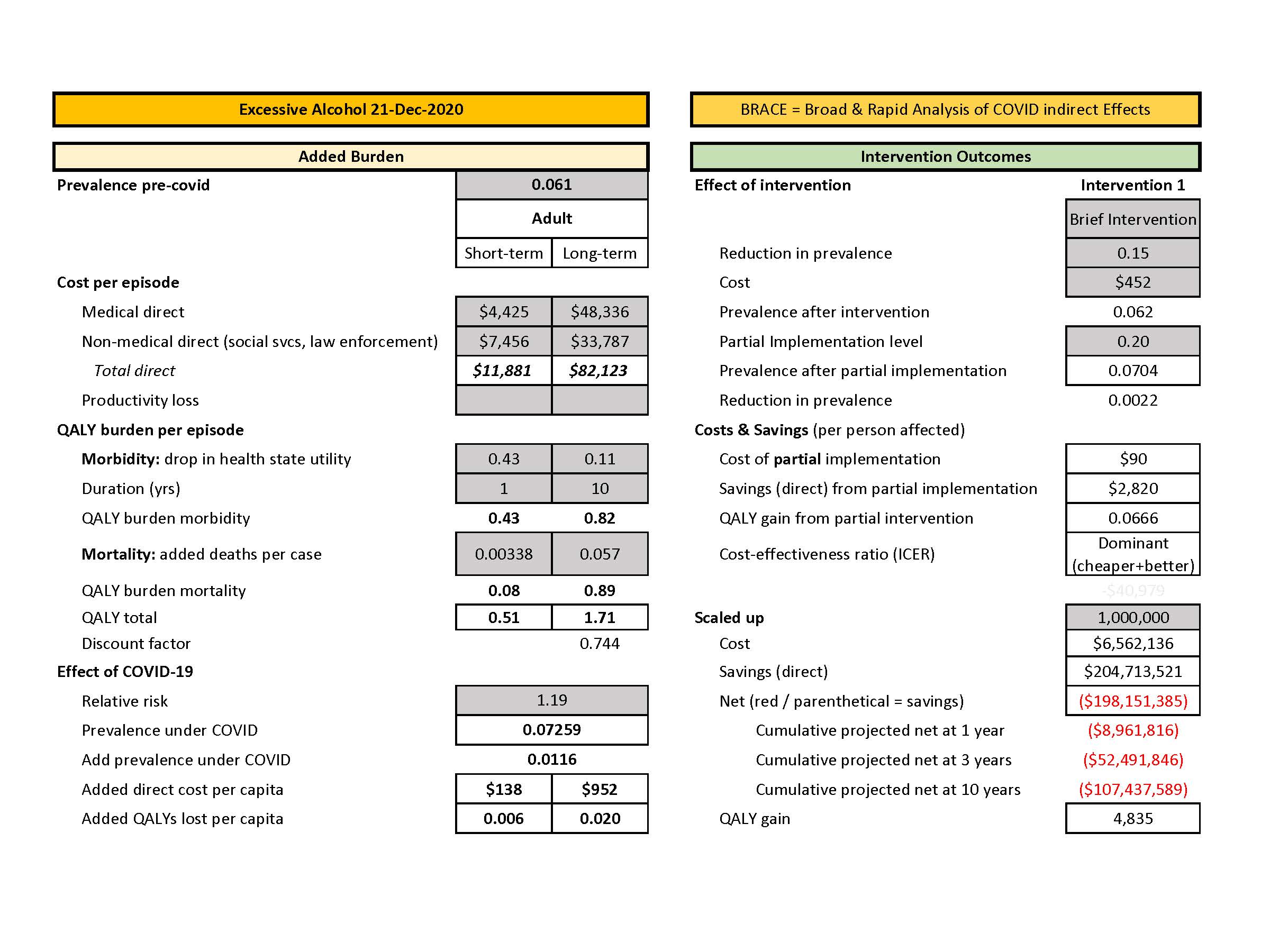
**

**Figure A9. One-way sensitivity analyses for net savings with screening and brief intervention for excessive alcohol use**


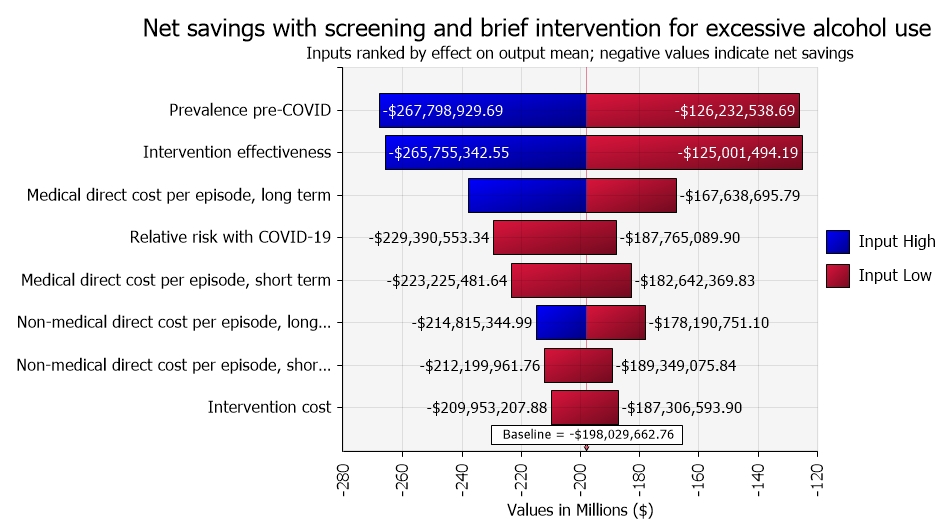


The intervention appeared to yield net savings $126-267 million per million population in one-way SAs.

**Figure A10. One-way sensitivity analyses for quality-adjusted life years (QALYs) gained with screening and brief intervention for excessive alcohol use**


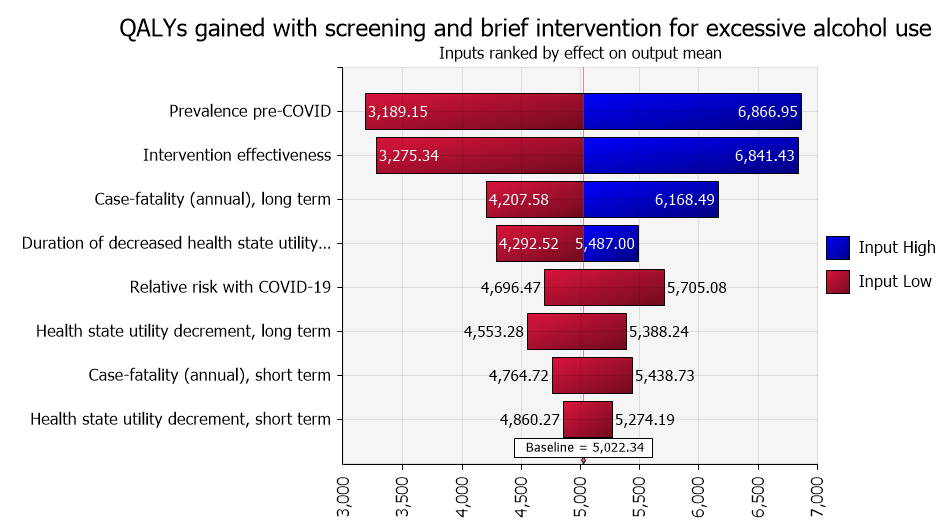


QALY gain ranged from 3,200 to 6,800 per million population in one-way SAs.

***Model parameters***

**Table A10. Inputs, uncertainty ranges, and sources for excessive alcohol use BRACE model**

| **Input** | **Base-case value** | **Uncertainty range** | **Sources and remarks** |
| --- | --- | --- | --- |
| Prevalence before COVID-19 | 6.1% | 3.1% - 9.2% | Substance Abuse and Mental Health Services Administration 2019 |
| Risk ratio for prevalence after COVID-19 | 1.19 | 1.07 – 1.42 | Pollard 2020, Smalley 2020 |
| ***Costs**** | | | |
| Short-term medical | $4,425 | $1,084 - $7,766 | Banta 2009, Barbosa 2015, Rosen 2008. Estimated medical cost attributed to alcohol for binge drinkers. |
| Long-term medical | $64,968 | $32,484 - $97,451 | Palmer 2000. Lifetime cost of diseases associated with alcohol addiction, and assuming 75% relapse among those who abused alcohol during the pandemic. |
| Short-term non-medical | $7,457 | $1,118 - $13,795 | Banta 2009, Barbosa 2015, Rosen 2008. Includes alcohol-related crimes, motor vehicles crashes, fire and destruction, and social welfare administration costs. |
| Long-term non-medical | $45,413 | $29,925 - $60,900 | American Addiction Centers . Lifetime cost of purchasing alcohol for abuse, assuming 75% relapse among those who abused alcohol during the pandemic and 70% of purchases are abused. |
| ***Health*** | | | |
| Short-term health state utility decrement | 0.43 | 0.38 – 0.48 | Kraemer 2005 |
| Long-term health state utility decrement | 0.11 | 0.08 – 0.14 | Kraemer 2005 |
| Short-term mortality | 0.34% | 0.33% - 0.34% | Chisholm 2004 |
| Long-term mortality | 5.7% | 2.9% - 12.8% | Costello 2006 |
| ***Intervention: Medication-assisted treatment*** | | | |
| Cost | $452 | $246 - $2,383 | Barbosa 2016 |
| Effectiveness | 15% | 7.5% - 22.5% | Bertholet 2005 |

*Costs are in 2020 US dollars. Long-term costs are presented before discounting.

**Stroke mortality**

Due to COVID-19, in-hospital mortality for patients admitted with stroke symptoms have increased an estimated 53%, to 0.02 (i.e., the likelihood of dying in hospital if presenting with a stroke). In Table A11, the prevalence shown (0.00015) was adjusted for the likelihood of hospital admission for stroke. The cost per case was $17,000 and the COVID-19-associated added direct cost per capita was just below $1. A public awareness campaign encouraging people to seek care if they experience symptoms was estimated to cost nearly $100 per person at risk for stroke and had an estimated 11% effectiveness in reducing consequences. Rolled out to a population of one million, this intervention was projected to cost $14,000 and led to 388 QALYs gained. The savings (averted direct costs) were estimated at $270,000, with net savings of $148,000 within a year and $242,000 by 10 years. The intervention remained dominant even with an efficacy one-tenth as much, or when campaign reach was halved. If only 30% of short-term costs were incurred in year 1 (as opposed to 60% in base case), year one net savings would be reduced to $66,600. If this value was instead 90%, $228,000 net savings would be achieved within one year.

**Table A11. BRACE results for effects of COVID-19 on stroke mortality**

**
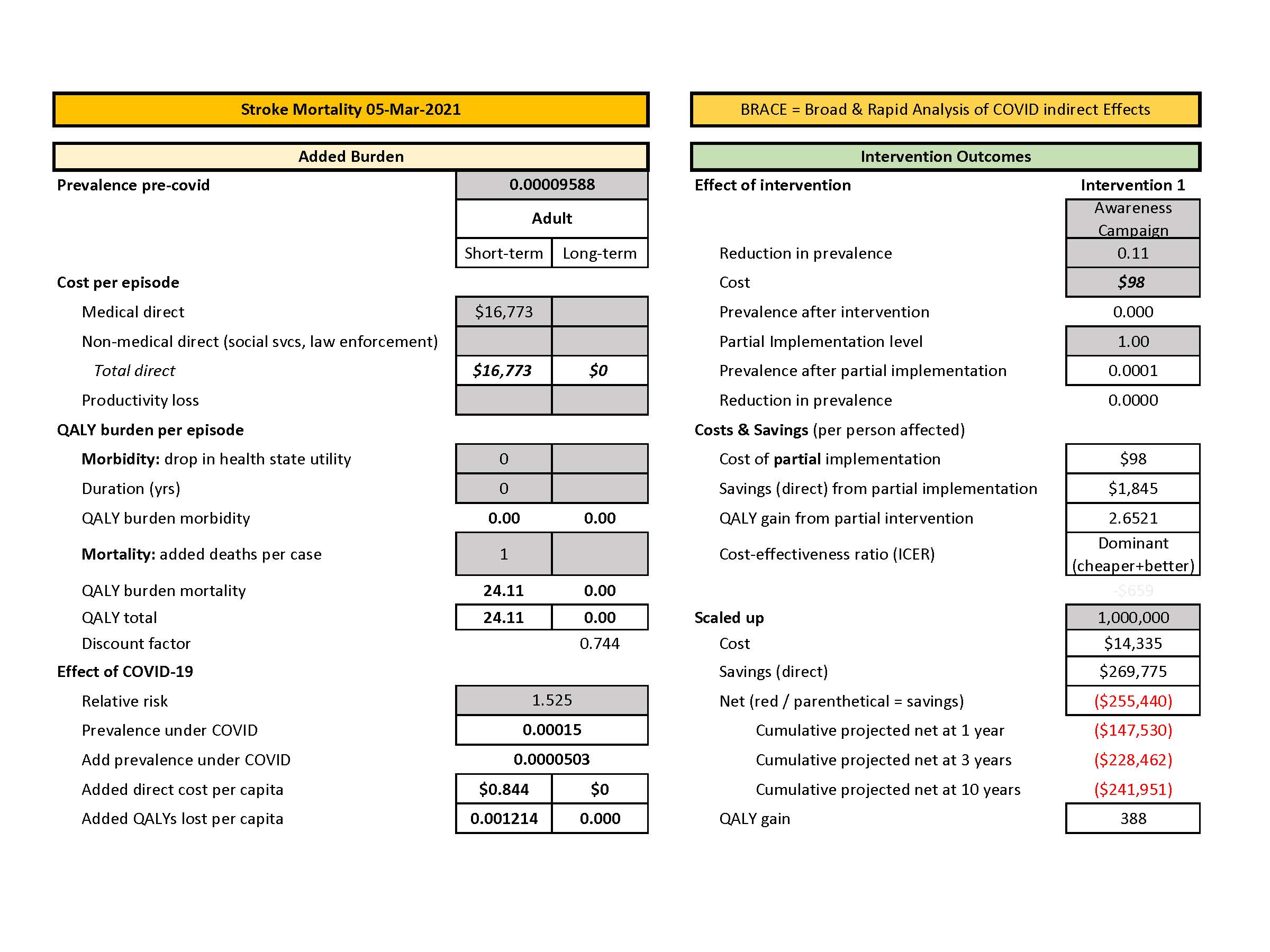
**

**Figure A11. One-way sensitivity analyses for net savings with public awareness campaign for stroke mortality**


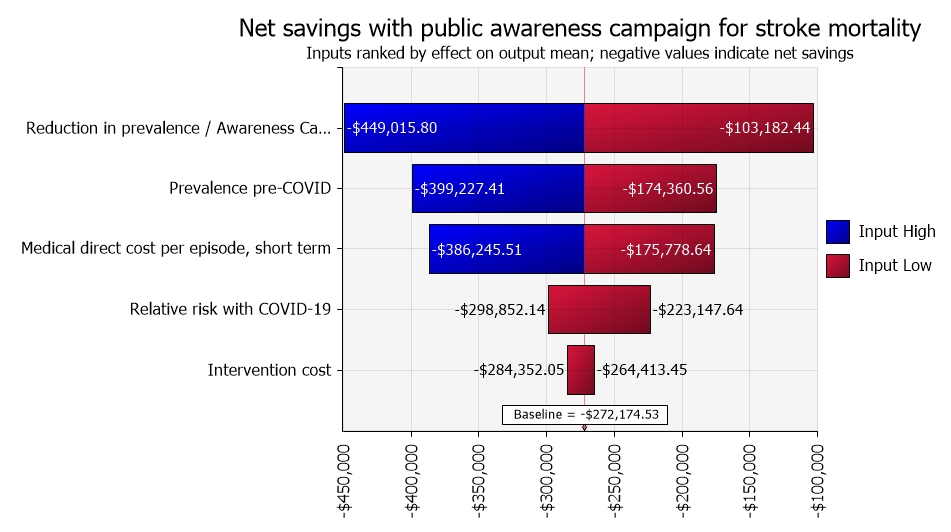


The intervention appeared to yield net savings between $103,000 to $449,000 per million population in one-way SAs.

**Figure A12. One-way sensitivity analyses for quality-adjusted life years (QALYs) gained with public awareness campaign for stroke mortality**


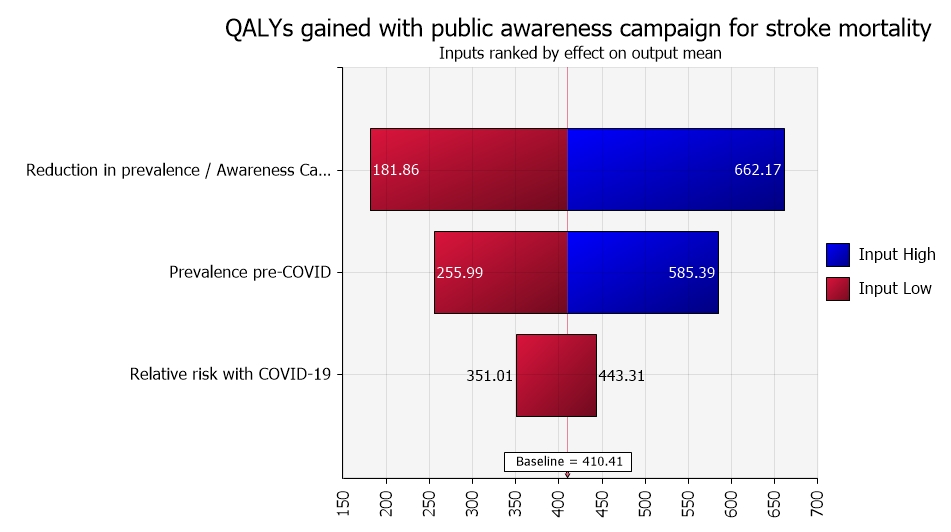


QALY gain ranged from 180 to 660 per million population in one-way SAs.

**Table A12. Inputs, uncertainty ranges, and sources for stroke mortality BRACE model**

| **Input** | **Base-case value** | **Uncertainty range** | **Sources and remarks** |
| --- | --- | --- | --- |
| Prevalence before COVID-19 | 0.037% | 0.019% - 0.056% | National Center for Health Statistics 2020. Stroke mortality rate. |
| Risk ratio for prevalence after COVID-19 | 1.53 | 1.40 – 1.67 | Agarwal 2020, Bhatt 2020, Friedlich 2021, Nagamine 2020, Nguyen-Huynh 2020, Siegler 2020 |
| ***Costs**** | | | |
| Short-term medical | $16,773 | $8,023 - $24,019 | Hernandez 2017. Cost of acute care. |
| ***Health*** | | | |
| Short-term health state utility decrement | 0 | 0 | The condition is death; the health utility is 0. |
| Short-term mortality | 100% | 100% | By definition. |
| ***Intervention: Public awareness advertisement campaign*** | | | |
| Cost | $98 | $49 - $147 | Cost per person at risk for stroke. |
| Effectiveness | 11% | 3% - 21% | Hodgson 2007 |

*Costs are in 2020 US dollars. Since the health condition is death, there are no non-medical and long-term costs.

**References**

1. Agarwal S, Scher E, Rossan-Raghunath N, Marolia D, Butnar M, Torres J, et al. Acute stroke care in a New York City comprehensive stroke center during the COVID-19 pandemic. J Stroke Cerebrovasc Dis. 2020;29(9):105068.

2. Ahmed O, Brockmeier D, Lee K, Chapman WC, Doyle MBM. Organ donation during the COVID-19 pandemic. American Journal of Transplantation. 2020;20(11):3081-8.

3. American Addiction Centers. The Cost of Drinking Alcohol: How Each U.S. City Compares [cited 2020 December 10]. Available from: <https://www.alcohol.org/guides/cost-of-drinking-alcohol-by-city/>.

4. Arroll B, Elley CR, Fishman T, Goodyear-Smith FA, Kenealy T, Blashki G, et al. Antidepressants versus placebo for depression in primary care. Cochrane Database Syst Rev. 2009(3):Cd007954.

5. Bansback N, Guh D, Ovieda-Joekes E, Brissette S, Harrison S, Janmohamed A, et al. Cost-effectiveness of hydromorphone for severe opioiduse disorder: findings from the SALOME randomized clinical trial. Addiction. 2018;113:1264-73.

6. Banta JE, Przekop P, Haviland MG, Pereau M. Binge Drinking Among California Adults: Results

from the 2005 California Health Interview Survey. The American Journal of Drug and Alcohol Abuse. 2009;34(6):801-9.

7. Barbosa C, Cowell A, Bray J, Aldridge A. The Cost-effectiveness of Alcohol Screening, Brief Intervention, and Referral to Treatment (SBIRT) in Emergency and Outpatient Medical Settings. Journal of Substance Abuse Treatment. 2015;53:1-8.

8. Barbosa C, Cowell AJ, Landwehr J, Dowd W, Bray JW. Cost of Screening, Brief Intervention, and Referral to Treatment in Health Care Settings. J Subst Abuse Treat. 2016;60:54-61.

9. Basu A, Kee R, Buchanan D, Sadowski LS. Comparative cost analysis of housing and case management program for chronically ill homeless adults compared to usual care. Health Serv Res. 2012;47(1 Pt 2):523-43.

10. Bertholet N, Daeppen JB, Wietlisbach V, Fleming M, Burnand B. Reduction of alcohol consumption by brief alcohol intervention in primary care: systematic review and meta-analysis. Arch Intern Med. 2005;165(9):986-95.

11. Bhatt AS, Moscone A, McElrath EE, Varshney AS, Claggett BL, Bhatt DL, et al. Fewer Hospitalizations for Acute Cardiovascular Conditions During the COVID-19 Pandemic. J Am Coll Cardiol. 2020;76(3):280-8.

12. Bullinger L, Carr J, Packham A. COVID-19 and crime: Effects of stay-at-home orders on domestic violence. Cambridge, MA: National Bureau of Economic Research; 2020 August 2020. Report No.: JEL No. I18,J12,K42.

13. California Deparment of Health Care Services. Medi-Cal Rates 2021 [cited 2021 March 8]. Available from: <https://files.medi-cal.ca.gov/rates/rateshome.aspx>.

14. Center for Behavioral Health Statistics and Quality. Results from the 2019 National Survey on Drug Use and Health: Detailed tables. Rockville, MD: Substance Abuse and Mental Health Services Administration; 2020.

15. Chandan JS, Thomas T, Bradbury‐Jones C, Taylor J, Bandyopadhyay S, Nirantharakumar K. Risk of Cardiometabolic Disease and All&#x2010;Cause Mortality in Female Survivors of Domestic Abuse. Journal of the American Heart Association. 2020;9(4):e014580.

16. Chisholm D, Rehm J, Van Ommeren M, Monteiro M. Reducing the global burden of hazardous alcohol use: a comparative cost-effectiveness analysis. J Stud Alcohol. 2004;65(6):782-93.

17. Conner A, Azrael D, Miller M. Suicide Case-Fatality Rates in the United States, 2007 to 2014. Annals of Internal Medicine. 2019;171(12):885-95.

18. Costello RM. Long-term mortality from alcoholism: a descriptive analysis. J Stud Alcohol. 2006;67(5):694-9.

19. Ferrari G, Agnew-Davies R, Bailey J, Howard L, Howarth E, Peters TJ, et al. Domestic violence and mental health: a cross-sectional survey of women seeking help from domestic violence support services. Global Health Action. 2014;7(1):25519.

20. Florence C, Luo F, Rice K. The economic burden of opioid use disorder and fatal opioid overdose in the United States, 2017. Drug and Alcohol Dependence. 2021;218:108350.

21. Friedlich D, Newman T, Bricker S. Impact of the COVID-19 Pandemic on Stroke Epidemiology and Clinical Stroke Practice in the US. J Stroke Cerebrovasc Dis. 2021;30(4):105639.

22. Glober N, Mohler G, Huynh P, Arkins T, O'Donnell D, Carter J, et al. Impact of COVID-19 Pandemic on Drug Overdoses in Indianapolis. J Urban Health. 2020;97(6):802-7.

23. Greenberg PE, Fournier A-A, Sisitsky T, Pike CT, Kessler RC. The Economic Burden of Adults With Major Depressive Disorder in the United States (2005 and 2010). The Journal of Clinical Psychiatry. 2015;75(2):155-62.

24. Gubits D, Shinn M, Wood M, Bell S, Dastrup S, Solari C, et al. Family Options Study: 3-Year Impacts of Housing and Services Interventions for Homeless Families. SSRN. 2016.

25. Hasin DS, Sarvet AL, Meyers JL, Saha TD, Ruan WJ, Stohl M, et al. Epidemiology of Adult DSM-5 Major Depressive Disorder and Its Specifiers in the United States. JAMA Psychiatry. 2018;75(4):336-46.

26. Hernandez I, Smith KJ, Zhang Y. Cost-effectiveness of non-vitamin K antagonist oral anticoagulants for stroke prevention in patients with atrial fibrillation at high risk of bleeding and normal kidney function. Thrombosis Research. 2017;150:123-30.

27. Hodgson C, Lindsay P, Rubini F. Can mass media influence emergency department visits for stroke? Stroke. 2007;38(7):2115-22.

28. Institute for Children, Poverty & Homelessness. Issue 2: Macroeconomic Causes of Family Homelessness. 2013.

29. Kirson NY, Scarpati LM, Enloe CJ, Dincer AP, Birnbaum HG, Mayne TJ. The Economic Burden of Opioid Abuse: Updated Findings. Journal of Managed Care & Specialty Pharmacy. 2017;23(4):427-45.

30. Kozloff N, Pinto AD, Stergiopoulos V, Hwang SW, O'Campo P, Bayoumi AM. Convergent validity of the EQ-5D-3L in a randomized-controlled trial of the Housing First model. BMC Health Serv Res. 2019;19(1):482-.

31. Kraemer KL, Roberts MS, Horton NJ, Palfai T, Samet JH, Freedner N, et al. Health utility ratings for a spectrum of alcohol-related health states. Med Care. 2005;43(6):541-50.

32. Krawczyk N, Eisenberg M, Schneider KE, Richards TM, Lyons BC, Jackson K, et al. Predictors of Overdose Death Among High-Risk Emergency Department Patients With Substance-Related Encounters: A Data Linkage Cohort Study. Annals of emergency medicine. 2020;75(1):1-12.

33. Krebs E, Enns B, Evans E, Urada D, Anglin D, Rawson RA, et al. Cost-Effectiveness of Publicly Funded Treatment of Opioid Use Disorder in California. Annals of Internal Medicine. 2018;168(1):10-9.

34. Larimer ME, Malone DK, Garner MD, Atkins DC, Burlingham B, Lonczak HS, et al. Health care and public service use and costs before and after provision of housing for chronically homeless persons with severe alcohol problems. Jama. 2009;301(13):1349-57.

35. Larochelle MR, Wakeman SE, Ameli O, Chaisson CE, McPheeters JT, Crown WH, et al. Relative Cost Differences of Initial Treatment Strategies for Newly Diagnosed Opioid Use Disorder: A Cohort Study. Med Care. 2020;58(10):919-26.

36. Los Angeles County Department of Public Health, Center for Health Impact Evaluation. Recent Trends In Mortality Rates and Causes of Death Among People Experiencing Homelessness in Los Angeles County. Los Angeles County Department of Public Health, Center for Health Impact Evaluation; 2019 October.

37. Ma J, Bao YP, Wang RJ, Su MF, Liu MX, Li JQ, et al. Effects of medication-assisted treatment on mortality among opioids users: a systematic review and meta-analysis. Mol Psychiatry. 2019;24(12):1868-83.

38. McLaughlin T. Using Common Themes: Cost-Effectiveness of Permanent Supported Housing for People With Mental Illness. Research on Social Work Practice. 2011;21(4):404-11.

39. Miller TR. Projected Outcomes of Nurse-Family Partnership Home Visitation During 1996–2013, USA. Prevention Science. 2015;16(6):765-77.

40. Mohler G, Bertozzi AL, Carter J, Short MB, Sledge D, Tita GE, et al. Impact of social distancing during COVID-19 pandemic on crime in Los Angeles and Indianapolis. J Crim Justice. 2020;68:101692.

41. Nagamine M, Chow DS, Chang PD, Boden-Albala B, Yu W, Soun JE. Impact of COVID-19 on Acute Stroke Presentation at a Comprehensive Stroke Center. Front Neurol. 2020;11:850.

42. Stroke Mortality by State [Internet]. Centers for Disease Control and Prevention. 2020 [cited January 10, 2021]. Available from: <https://www.cdc.gov/nchs/pressroom/sosmap/stroke_mortality/stroke.htm>.

43. Nguyen-Huynh MN, Tang XN, Vinson DR, Flint AC, Alexander JG, Meighan M, et al. Acute Stroke Presentation, Care, and Outcomes in Community Hospitals in Northern California During the COVID-19 Pandemic. Stroke. 2020;51(10):2918-24.

44. Olfson M, Amos TB, Benson C, McRae J, Marcus SC. Prospective Service Use and Health Care Costs of Medicaid Beneficiaries with Treatment-Resistant Depression. Journal of Managed Care & Specialty Pharmacy. 2018;24(3):226-36.

45. Palmer AJ, Neeser K, Weiss C, Brandt A, Comte S, Fox M. The Long-Term Cost-Effectiveness of Improving Alcohol Abstinence with Adjuvant Acamprosate. Alcohol and Alcoholism. 2000;35(5):478-92.

46. Peterson C, Kearns MC, McIntosh WL, Estefan LF, Nicolaidis C, McCollister KE, et al. Lifetime Economic Burden of Intimate Partner Violence Among U.S. Adults. Am J Prev Med. 2018;55(4):433-44.

47. Pierce M, Hope H, Ford T, Hatch S, Hotopf M, John A, et al. Mental health before and during the COVID-19 pandemic: a longitudinal probability sample survey of the UK population. Lancet Psychiatry. 2020;7(10):883-92.

48. Piquero AR, Riddell JR, Bishopp SA, Narvey C, Reid JA, Piquero NL. Staying Home, Staying Safe? A Short-Term Analysis of COVID-19 on Dallas Domestic Violence. Am J Crim Justice. 2020:1-35.

49. Pollard MS, Tucker JS, Green HD, Jr. Changes in Adult Alcohol Use and Consequences During the COVID-19 Pandemic in the US. JAMA Netw Open. 2020;3(9):e2022942-e.

50. Rosen SM, Miller TR, Simon M. The Cost of Alcohol in California. Alcoholism: Clinical and Experimental Research. 2008;32(11):1925-36.

51. Siegler JE, Heslin ME, Thau L, Smith A, Jovin TG. Falling stroke rates during COVID-19 pandemic at a comprehensive stroke center. J Stroke Cerebrovasc Dis. 2020;29(8):104953.

52. Smalley CM, Malone DA, Jr., Meldon SW, Borden BL, Simon EL, Muir MR, et al. The impact of COVID-19 on suicidal ideation and alcohol presentations to emergency departments in a large healthcare system. The American Journal of Emergency Medicine. 2020.

53. Smith S, Zhang X, Basile K, Merrick M, Wang J, Kresnow M, et al. The National Intimate Partner and Sexual Violence Survey (NISVS): 2015 Data Brief - Updated Release. Atlanta, GA: National Center for Injurt Prevention and Control, Centers for Disease Control and Prevention; 2018.

54. Sobocki P, Ekman M, Agren H, Krakau I, Runeson B, Mårtensson B, et al. Health-related quality of life measured with EQ-5D in patients treated for depression in primary care. Value Health. 2007;10(2):153-60.

55. Substance Abuse and Mental Health Services Administration. Behavioral Health Barometer: United States, Volume 5: Indicators as measured through the 2017 National Survey on Drug Use and Health and the National Survey of Substance Abuse Treatment Services. Rockville, MD: Substance Abuse and Mental Health Services Administration; 2019. Contract No.: SMA–19–Baro-17-US.

56. The Council of Economic Advisors. The underestimated cost of the opioid crisis. 2017.

57. Twenge JM, Joiner TE. U.S. Census Bureau-assessed prevalence of anxiety and depressive symptoms in 2019 and during the 2020 COVID-19 pandemic. Depression and Anxiety. 2020;37(10):954-6.

58. QuickFacts: California [Internet]. 2020 [cited November 21, 2020]. Available from: <https://www.census.gov/quickfacts/CA>.

59. California Homelessness Statistics [Internet]. 2020 [cited November 21, 2020]. Available from: <https://www.usich.gov/homelessness-statistics/ca/>.

60. Villaroel MA, Terlizzi EP. Symptoms of Depression Among Adults: United States, 2019. NCHS Data Brief. Hyattsville, MD: National Center for Health Statistics; 2020.

61. Wittenberg E, Bray JW, Aden B, Gebremariam A, Nosyk B, Schackman BR. Measuring benefits of opioid misuse treatment for economic evaluation: health-related quality of life of opioid-dependent individuals and their spouses as assessed by a sample of the US population. Addiction. 2016;111(4):675-84.

62. Wittenberg E, Lichter EL, Ganz ML, McCloskey LA. Community Preferences for Health States Associated with Intimate Partner Violence. Medical Care. 2006;44(8):738-44.

63. Wright BJ, Vartanian KB, Li H-F, Royal N, Matson JK. Formerly Homeless People Had Lower Overall Health Care Expenditures After Moving Into Supportive Housing. Health Affairs. 2016;35(1):20-7.

64. Wu F, Stevens M. The Services Homeless Single Adults Use and their Associated Costs: An Examination of Utilization Patterns and Expenditures in Los Angeles County over One Fiscal Year. 2016.

65. Wu J, Dean KS, Rosen Z, Muennig PA. The Cost-effectiveness Analysis of Nurse-Family Partnership in the United States. Journal of Health Care for the Poor and Underserved. 2017;28(4):1578-97.

66. Zaretzky K, Flatau P, Clear A, Conroy E, Burns L, Spicer B. The cost of homelessness and the net benefit of homelessness programs: a national study. Melbourne, Australia: Australian Housing and Urban Research Institute; 2013.
