# Supplementary material for: Indirect COVID-19 health effects and potential mitigating interventions: Cost-effectiveness framework
Source: PLoS One. 2022 Jul 18;17(7):e0271523. doi: 10.1371/journal.pone.0271523 (PMC9292069; doi:10.1371/journal.pone.0271523)
Supplement: S2 File — (DOCX) [file pone.0271523.s002.docx]

**Supplement B: Key findings of literature review and assessment of strength of evidence**

This section describes our methodology for assessing the strength of evidence for the change in health conditions during the pandemic. The strength of evidence for selected health conditions are presented in Table B1.

We assessed the strength of the evidence by considering five domains: 1) data quantity; 2) external validity [with three sub-domains]; 3) risk of bias [with two sub-domains]; 4) precision; and 5) consistency. The intention of this is to provide a high-level assessment of the trustworthiness of evidence for high level decision-making in an expedited manner, and we have intentionally left out a more nuanced assessment of the internal and external validity that require more intensive review of the literature, often dual-independent review by highly trained individuals in the subject matter.

**1. Data quantity/Evidence of replicability:*** Number of studies or unique data points contributing to the estimate for each outcome.

- **High:** 4 or more
- **Moderate:** 2-3
- **Low:** 1

**2. External validity (direct versus indirect evidence):**** We assessed external validity through consideration of three sub-domains, listed below (2a-2c):

**a. Population/setting applicability:** How well do the settings and populations of included studies match those of California?

- **High:** At least one California data point and the vast majority (as a rule of thumb, 75%-80% or more) of all data coming from the US
- **Moderate:** No California data but the vast majority of data coming from the US
- **Low:** Vast majority of data coming from countries other than the US [Note: these data were selectively included if CA/US data not available]

**b. Contextual relevance:** How well does the identified evidence reflect the COVID-19 era?

- **High:** COVID-19 era data
- **Moderate:** Pre-COVID-19 era data but sufficient contextual similarities
- **Low:** Pre-COVID-19 era data, but unclear contextual similarities [Note: we selectively included these data as a placeholder when no other contextually relevant data were available]

**c. Outcome measure relevance:** How well does the included indicator predict the intended outcome? For example, the extent to which high blood pressure (the reported indicator) may be a proxy for myocardial infarction (the intended outcome); or to which police calls may be a proxy for substantiated domestic violence.

- **High:** Reported indicator and intended outcome are the same
- **Moderate:** Reported indicator is a strong proxy for the intended outcome
- **Low:** Reported indicator is a weak proxy for the intended outcome

***Overall external validity score (based on 2a-2c):***

- **High:** If at least one of 2a-2c scored “High” and none of 2a-2c are scored “Low.”
- **Moderate:** If all are scored “Moderate”; OR if one is scored “High,” one “Moderate” and one “Low”; OR if two are scored “High” and one is scored “Low.”
- **Low:** None of the above

**3. Risk of bias (internal validity):**** We assessed risk of bias through consideration of two sub-domains (3a-3b):

**a. Outcome measurement.** What is the extent to which subjectivity of the outcome can affect the validity of the results? For example, measures like “number of ED visits” from hospital records are more objective than self-reported non-standard mental health measures.

- **High:** Objective/hard outcome (e.g., number of emergency department visits, or stroke): easy to quantify
- **Moderate:** Subjective outcome measured via a standardized or widely recognized tool (e.g., depression, anxiety)
- **Low:** Subjective outcome without a clear indication of how the outcome was measured

**b. Assessment of baseline risk:** What study methodology was used in obtaining the baseline and outcome data, and how similar are the two groups being compared? For example, did the study take raw data from 2019 and compare it to 2020 data, or did they do some statistical adjustments? Are the participants in baseline data similar to the later (e.g., COVID-19 era) participants?

- **High:** At least one study with baseline data from the same cohort or population; others may include data from different cohorts but with reasonable comparison groups
- **Moderate:** No cohort data of the same population, but vast majority of studies with baseline data from a different cohort (e.g., serial cross-sections); with reasonable comparators and using consistent survey methods and/or advanced statistical adjustments
- **Low:** Vast majority of studies with baseline data from different populations / studies

***Overall internal validity score (based on 3a-3b):***

- **High:** If at least either 3a-3b scored “High” and none of 2a-2c are scored “Low”
- **Moderate:** If both scored “Moderate”; OR if one is scored “Low” and the other is “High.”
- **Low:** None of the above

**4. Uncertainty/precision:*** Effect sizes with tighter confidence intervals (CI; or range) were preferred over those with wide CIs, or those with no CIs.

- **High:** Tight CIs (range < 0.4)
- **Moderate:** Wide CIs (range > 0.40) but not crossing the null
- **Low:** Wide CIs AND crossing the null; OR no CI reported

**5. Consistency/statistical heterogeneity:*** What is the extent to which data points are consistent in the direction and overall magnitude of the effect?

- **High:** Vast majority (> 75%) of data points in the same direction
- **Moderate:** More than half (50%-74%) of data points in the same direction
- **Low:** Data points are highly heterogeneous in regard to direction and magnitude of effect

**Note:** If there is only a single data point, we rate it as “High.”

***Overall Strength of Evidence score based on 1-5, above:***

- **High:** If no “Low” and at least one “High”
- **Moderate:** All “Moderate” OR no more than one is “Low”
- **Low:** Two are “Low”
- **Very low:** Three or more are “Low”

Note: If #3 (Risk of bias) is “Low” for studies scored Moderate or Low, downgrade one level

*Domain assessed at the aggregate level only

** Data extracted at individual data point to help assess overall scoring.

**Table B1. Brief key findings from the literature review for health conditions worsened during the COVID-19 pandemic**

| Public Health Condition | Key Findings | Confidence |
| --- | --- | --- |
| Mental health (16) | Large increase in rates and severity of depression and anxiety as well as pediatric behavioral complaints | **HIGH** |
| Stroke (14) | Large decrease in presentation of patients with stroke and large increase in worsened discharge outcomes, including stroke mortality | **HIGH** |
| Intimate partner violence (7) | Large increase in reports of intimate partner violence | **HIGH** |
| Social determinants of health (7) | Increased food insecurity, job loss | **HIGH** (food insecurity) to **VERY LOW** (homelessness, job loss) |
| Substance use (7) | Increased substance use and opioid-related emergency response | **MODERATE** |
