# Supplementary material for: Indirect COVID-19 health effects and potential mitigating interventions: Cost-effectiveness framework
Source: PLoS One. 2022 Jul 18;17(7):e0271523. doi: 10.1371/journal.pone.0271523 (PMC9292069; doi:10.1371/journal.pone.0271523)
Supplement: S3 File — (DOCX) [file pone.0271523.s003.docx]

**Supplement C: Selection of COVID-19 indirect health impacts and mitigation strategies**

**1) Selection of health condition to be included in BRACE**

A systematic review yielded 93 articles that were eligible for data synthesis as they reported a range of significantly worsened mental and physical health outcomes and significantly worsened social determinants of health associated with the COVID-19 pandemic. Table C1 provides a summary of criteria used to make decisions about which health conditions to subject to the economic forecast modeling. Rows in the table represent a broad range of indirect health outcomes that could theoretically be affected by the pandemic. Along the rows, we show a combination of criteria used to narrow down the list of possible indirect health outcomes to a feasible number.

**Table C1: RAPID priority-setting decision matrix**

| Public Health Condition | # of  Studies | Change in Prevalence Due to COVID-19   *Relative Risk* | Life Years Lost (YLL) due to the Condition    *YLL per 100,000* | Disability-Adjusted Life Years Lost due to Condition (DALYs) *per 100,000, both sexes, all ages* | Association with 4+ Adverse Childhood Experiences (ACEs)  *Odds Ratio for Adults* |
| --- | --- | --- | --- | --- | --- |
| *Mental health problems (e.g., mood or anxiety disorders) | 17 | Increased | 6.4 | 701.8 [depressive disorders]  583.6 [anxiety disorders] | 3.7 [anxiety] 4.7 [depression] |
| Heart disease | 9 | Presentations decreased,  severity increased | 121.9 [hypertensive]  347.9 [Ischemic] | 2,061 [Ischemic heart disease] | 2.1 [cardiovascular disease] |
| *Stroke | 9 | Presentations decreased,  severity increased | 138.4 | 935.3 | 2.1 [cardiovascular disease] |
| Acute stress, fear, and loneliness | 8 | Increased | _ | _ | 3.7 [stress] |
| *Intimate partner violence (IPV) | 7 | Increased | 1.5 per episode | 369.5 [IPV] | 7.5 [violence victimization] 8.1 [violence perpetration] |
| *Alcohol and drug consumption | 7 | Increased | 655 [alcohol] 1232 tobacco] 110 [illicit drugs] | 1,278.5 [drug use disorders]  405.7 [alcohol use disorders] | 6.8 [problematic alcohol use] 10.2 [problematic drug use] |
| Suicide/self-harm | 7 | Increase in suicidal ideation,  suicide prevalence not yet clear | 304.5 | 463.8 [self-harm] | 37.5 [suicide attempt] 10.5 [suicidal ideation] |
| Dietary and exercise habits | 6 | Decreased | _ | _ | 1.1 [physical inactivity] |
| Emergency care use | 5 | Decreased | _ | _ | 2.8 [high ER use] |
| Homicide/crime | 4 | Reduction in calls for service,  projected increase in gun injuries | 190.4 | 364.9 [Interpersonal violence] | _ |
| Diabetes mellitus | 4 | Unknown | 105.4 | 1,099.1 [diabetes] | 1.4 |
| Child maltreatment | 2 | Increase in rate in UK and severity in US (one study each) | **_** | **_** | **_** |
| Organ and blood donation | 2 | Decreased | _ | _ | _ |
| Communicable diseases | 2 | Vaccinations decreased | 49.4 [other infectious diseases, excluding TB] | _ | _ |
| Chronic respiratory disease | 2 | Presentations decreased | 474.1 | 1,083.9 [COPD]  413.4 [asthma] | 3.1 (2.5 – 3.8) |
| Musculoskeletal disorders | 1 | Presentations decreased | 41.9 | 1,040.6 [other musculoskeletal disorders] | _ |
| Food insecurity | 1 | Increased | _ | _ | _ |
| Cancer | 1 | Access to / use of care decreased | 920.2 [all cancers] | 774.5 [lung cancer] | 2.3 |
| Hypertension | 0 | Unknown | _ | _ | _ |
| Neurological disorders | 0 | Unknown | 364.7 | 727.0 [headache disorders]  525.1 [Alzheimer’s diseases and other dementias] | _ |
| Digestive diseases | 0 | Unknown | 64.8 |  | 2.8 |

**Table C1, continued**

| Public Health Condition | # of  Studies | Change in Prevalence Due to COVID-19   *Relative Risk* | Life Years Lost (YLL) due to the Condition    *YLL per 100,000* | Disability-Adjusted Life Years Lost due to Condition (DALYs) *per 100,000, both sexes, all ages* | Association with 4+ Adverse Childhood Experiences (ACEs)  *Odds Ratio for Adults* |
| --- | --- | --- | --- | --- | --- |
| Substance use disorders | 0 | Unknown | 624.4 | 1,278.5 [drug use disorders] | >2 [ drug use] |
| *Housing insecurity | 0 | Unknown but projected to increase | 345 potential-YLL per 1,000 person-years of observation | _ | 3.9 |
| Unintentional injuries | 0 | Unknown | 245.3 | 630.1 [road injuries]  540.2 [falls] | 2.6 |
| Maternal/neonatal diseases | 0 | Unknown | 395.6 | 362.7 [neonatal disorders]  16.2 [maternal disorders | _ |
| Skin and subcutaneous disorders | 0 | Unknown | 15.7 | 191.1 [dermatitis]  116.7 [viral skin diseases]  77.9 [other skin and subcutaneous diseases] | _ |
| Nutritional diseases | 0 | Unknown | 7.4 | 51.0 [dietary iron deficiency]  34.5 [protein-energy malnutrition]  10.5 [other nutritional deficiencies] | _ |

Key:

- = there was not enough information to fill in cell.

* = conditions selected for the BRACE model.

# Studies: The number of COVID-19-era studies published and gray-literature studies showing changes in rates of the condition during the pandemic.

Increase Under COVID-19 (RR): If available in the literature, the relative risk of the condition after the pandemic as compared to pre-COVID-19.

Ranked Cause of Death in CA: Rank in causes of death for Californians as estimated by Institute for Health Metrics and Evaluation (IHME), 2017.

Years of Life Lost per 100K: Years of life lost due to the condition as estimated by California Community Burden of Disease and Cost Engine.

Association with 4+ ACEs: Degree of correlation between condition and ACEs based on reviews of the literature.

Sources for YLL: California Department of Public Health, 2021; Hibbs, 1994; Institute for Health Metrics and Evaluation, 2021; Single, 1999

Sources for OR for 4+ ACEs: Chartier, 2010; Hughes, 2017; Miller, 2020

Sources for DALYs: IHME Global burden of disease

**2) Selection of mitigation strategies**

Data on the impact of COVID-19 were chosen primarily based on the strength of evidence and relevance to the California context. Additional considerations were necessary when selecting mitigation strategies that the State of California might consider implementing, which are shown in the table below. Recommendations for mitigation strategies were based on the following criteria:

1. Recommended during expert consultation: For each indirect health outcome, we consulted one or more scientific-content experts for guidance on commonly used, evidence-based interventions suitable to implement during and after the pandemic.
2. Peer-reviewed research showed that the mitigation strategy is both effective and cost-effective at the population level.
3. Statistical information in the peer-reviewed literature made it possible to calculate an improvement in relative risk due to the mitigation strategy—a necessary input for the BRACE model.
4. Implementation of the mitigation strategy would be possible under a range of pandemic conditions, including full sheltering-in-place. For example, certain interventions, such as the Blues Program, which is a cognitive-behavioral, group, depression-prevention program based in high schools (Rohde et al., 2014; Stice et al., 2010) might not be relevant in the context of COVID-19 since many schools paused in-person learning.
5. The mitigation strategy would directly affect the specific outcome input in the BRACE model. For example, Table 11 shows that six mitigation strategies were considered to address increases in intimate partner violence (IPV) during the pandemic. Although computer-based screening in Emergency Departments might increase detection of IPV (e.g., Trautman, 2007), this would not directly mitigate the occurrence of violence or address the sequelae of violence, which were the foci for IPV included in the BRACE model.

Table C2 depicts the interventions that were considered and for modeling and explains reasons for inclusion or exclusion. Table C3 contains the data that were extracted for selected interventions.

**Table C2: Potential Mitigation Strategies for Priority Public Health Conditions and Rationale for Inclusion and Exclusion**

| Priority Public Health Condition | Mitigation Strategy | Include/ Exclude | Explanation |
| --- | --- | --- | --- |
| Intimate partner violence | Computer-based-screening in emergency departments (EDs) | Exclude | Effective but outcome doesn’t match pandemic changes |
|  | Brief advocacy and/or case management | Exclude | Confidence interval includes 1 |
|  | Cognitive-behavioral therapy | Exclude | Excluded based on discussions with expert advisors; lack of published papers. |
|  | Expect Respect school-based prevention | Exclude | Effective, but population does not match (teens vs. general population) |
|  | **Nurse-Family Partnership Home Visiting Program** | **Include** | **Effective based on data from randomized-controlled trial; several US studies available.** |
|  | MyPlan App | Exclude | No improvement in IPV |
| Depression | The Blues Program | Exclude | Effective, but population does not match, might not be applicable during COVID-19 |
|  | **Cognitive-behavioral therapy (with SSRIs)** | **Include** | **Effective, plenty of supportive data from meta-analyses, US studies available** |
| Excessive alcohol use | Alcohol taxes | Exclude | Could not calculate risk ratio |
|  | School-based prevention (Strengthening Families Program) | Exclude | No confidence interval reported; population does not match |
|  | **Brief intervention** (part of SBIRT -- screening, brief intervention, and referral to treatment) | **Include** | **Evidence of effectiveness from meta-analyses; supported by expert advisors** |
| Opioid use disorder | Take-home Naloxone | Exclude | Outcomes measured do not match COVID-19 era outcomes reported |
|  | **Medication-assisted treatment** | **Include** | **Evidence of effectiveness from meta-analyses, supported by expert advisors** |
| Homelessness | High-intensity case management | Exclude | Confidence intervals include 1 |
|  | **Rent subsidies** | **Include** | **Evidence of effectiveness and ability to calculate costs from reliable data sources** |
| Stroke mortality* | **Public awareness campaign** | **Include** | **Effective, COVID-19 appropriate** |
|  | Home blood pressure monitoring | Exclude | Outcomes measured do not match COVID-19 era outcomes reported |

*Literature reviews suggested that COVID-19-era increases in stroke mortality were the result of delayed medical care-seeking. An appropriate mitigation strategy was therefore one that increased public awareness and early detection.

| **Table C3. Interventions Included in BRACE Modeling** |
| --- |

| Condition | Intervention | Outcome | Risk Ratio & 95% CI | Location | Author & Year |
| --- | --- | --- | --- | --- | --- |
| Depression | SSRI medications | Significant improvement (50% decrease in score) | RR 0.75 (0.62 – 0.90) | USA/Europe (meta-analysis) | Arroll 2009 |
| Depression | Cognitive-Behavioral Therapy (CBT) | Remission of depression | RR 0.65  (0.51 – 0.83) | Worldwide (meta-analysis) | Cuijpers 2014 |
| Intimate partner violence | Nurse-Family Partnership Home Visiting | IPV within 4 years | RR 0.79 (0.99 – 0.64) | USA | Miller 2015 |
| Homelessness | Rent subsidies | # of households receiving housing vouchers | 6.67 (NA) | USA | ICPH  2013 |
| Opioid use disorder | Medication-Assisted Treatment (MAT) | Overdose mortality during treatment | RR 0.12 (0.07 – 0.22) | Worldwide (meta-analysis) | Ma 2019 |
| Opioid use disorder | Medication-Assisted Treatment (MAT) | All-cause mortality during treatment | RR 0.39 (0.26 – 0.58) | Worldwide (meta-analysis) | Ma 2019 |
| Excessive alcohol use | Brief intervention (as in SBIRT) | Alcohol consumption | -50g of alcohol/week (-65 - -34); 15% greater decrease than controls | Worldwide (meta-analysis) | Bertholet 2005 |
| Stroke | Public awareness campaigns | ER stroke admissions | RR 1.07 (1.001 – 1.14) | England | Flynn 2014 |
| Stroke | Public awareness campaigns | ER stroke admissions within 5 hours of symptoms | RR 1.11  (1.03 – 1.21) | Canada | Hodgson 2007 |
